# Long-term ambient air pollution is not associated with circulating soluble urokinase plasminogen activator receptor

**DOI:** 10.1097/EE9.0000000000000532

**Published:** 2026-10-06

**Authors:** Bahjat Z. Ghazzal, Anis Ismail, Yiyuan Huang, Amanda Gassett, Theresa Farhat, Ramya Elangovan, Pennelope Blakely, Susan R. Heckbert, Joel D. Kaufman, Salim S. Hayek

**Affiliations:** a Department of Medicine, University of Massachusetts Chan Medical School, Worcester, Massachusetts; b Department of Internal Medicine, University of Texas Medical Branch, Galveston, Texas; c Department of Biostatistics, University of Michigan, Ann Arbor, Michigan; d Department of Environmental and Occupational Health Sciences, School of Public Health, University of Washington, Seattle, Washington; e Department of Epidemiology, School of Public Health, University of Washington, Seattle, Washington; f Department of Internal Medicine, Division of Cardiology, University of Michigan, Ann Arbor, Michigan

**Keywords:** Air pollution, Particulate matter, suPAR, Inflammation, Cardiovascular disease, Biomarker, Multi-Ethnic Study of Atherosclerosis, Epidemiology

## Abstract

**Background:**

Soluble urokinase plasminogen activator receptor (suPAR) is a stable chronic-inflammation biomarker causally implicated in atherosclerosis, yet its modifiable determinants remain largely uncharacterized. Long-term ambient air pollution is an established cardiovascular risk factor and a biologically plausible determinant of suPAR.

**Methods:**

In this cross-sectional analysis, we tested associations of long-term residential air pollution with circulating suPAR in 5,072 participants of the Multi-Ethnic Study of Atherosclerosis, drawn from six US metropolitan regions and enrolled during 2000–2002. Residential exposures were estimated with the validated Multi-Ethnic Study of Atherosclerosis Air spatiotemporal model, and serum suPAR was measured by enzyme-linked immunosorbent assay.

**Results:**

In multivariable linear regression adjusted for demographic, socioeconomic, and cardiovascular risk factors, neither fine particulate matter, nitrogen dioxide, nitrogen oxides, ozone, nor light-absorbing carbon was associated with suPAR; the fine particulate matter confidence interval excluded effect sizes of two percent or greater per 5 µg/m^3^.

**Conclusions:**

Together with two prior cohorts spanning two continents and three age ranges, these findings indicate that long-term ambient air pollution is unlikely to be a meaningful determinant of circulating suPAR or a major mediator of the air-pollution–cardiovascular-disease association, and suggest that the search for modifiable determinants of this clinically actionable biomarker should focus elsewhere.

**What this study adds**

The modifiable determinants of soluble urokinase plasminogen activator receptor (suPAR)—a stable biomarker causally linked to cardiovascular disease—are largely unknown. In the largest and most diverse cohort yet to examine the question (5,072 Multi-Ethnic Study of Atherosclerosis participants), long-term exposure to ambient air pollution was not associated with circulating suPAR, with confidence intervals that excluded even small effects. Together with two earlier cohorts, these results make ambient air pollution an unlikely determinant of suPAR and an unlikely mediator of its link to cardiovascular disease, redirecting the search for modifiable determinants elsewhere.

## Introduction

Soluble urokinase plasminogen activator receptor (suPAR) is a chronic-inflammation biomarker shed primarily from bone-marrow myeloid cells.^1^ Clinical, genetic, and experimental data support a causal role for suPAR in cardio-kidney metabolic diseases.^2^ Yet the genetic variants identified to date explain only a small fraction of interindividual variation in circulating suPAR,^3^ indicating that the bulk of population-level variance is driven by nongenetic factors that remain largely uncharacterized.

suPAR levels are generally stable with modest change over time (<10% in 5 years) and are not impacted by circadian rhythm or acute inflammatory stressors.^4^ Outside of cigarette smoking, which elevates suPAR by ~17% and reverses with cessation,^5^ few environmental determinants of suPAR are established. Long-term ambient air pollution is a well-documented cardiovascular risk factor, including within the Multi-Ethnic Study of Atherosclerosis (MESA),^6^ and if air pollution acts in part through chronic systemic inflammation and bone-marrow myeloid activation, circulating suPAR is a candidate mediator. Two prior cohorts have tested this hypothesis and reported null associations.^7,8^ We tested the hypothesis in 5,072 MESA participants, the largest and most ethnically and regionally diverse cohort to address this question. MESA spans six US metropolitan areas with substantial contrast in air pollution levels, sources, and composition, and exposure was quantified using a state-of-the-art spatiotemporal model.^9,10^

## Methods

We conducted a cross-sectional analysis of MESA participants at the baseline examination. MESA enrolled 6,814 adults aged 45–84 years free of clinical cardiovascular disease from six US metropolitan areas (Baltimore, Chicago, Forsyth County, NC, Los Angeles, New York, St. Paul) during 2000–2002.^11^ Long-term residential air pollution exposure was estimated by the validated MESA Air spatiotemporal model as 1-year averages before enrollment for fine particulate matter (PM_2.5_), nitrogen dioxide (NO_2_), nitrogen oxides (NO_x_), and ozone (O_3_), and as a calendar-year 2000 spatial-only baseline average for light-absorbing carbon (LAC, a black-carbon proxy).^9,10^ Serum suPAR was measured at enrollment in the Hayek laboratory by enzyme-linked immunosorbent assay (suPARnostic, ViroGates, Denmark).^3^ suPAR was log2-transformed to reduce right skew and to express associations as relative differences. We modeled log2(suPAR) as a function of each pollutant in multivariable linear regression, adjusted for age, sex, race/ethnicity, MESA site, body mass index, cystatin-C-based estimated glomerular filtration rate, smoking status (never/former/current) and pack-years, education, neighborhood socioeconomic status (census-tract principal component), systolic blood pressure, antihypertensive medication, low- and high-density lipoprotein cholesterol, statin use, and diabetes. Covariates comprise the established correlates of circulating suPAR in MESA together with the confounders used in MESA Air analyses of long-term residential exposure.^3,6,12^ All were ascertained at the baseline examination as previously described:^11^ age, sex, race/ethnicity, education, smoking status, pack-years and diabetes by standardized questionnaire; height, weight and seated systolic blood pressure by trained staff; medication use by review at the visit; and lipids and cystatin C in the central laboratory, with estimated glomerular filtration rate calculated from cystatin C using the Stevens equation, as in previous MESA analyses.^13^ Associations are reported as percent difference in suPAR per prespecified contrast (PM_2.5_ per 5 µg/m^3^, NO_x_ per 40 ppb, NO_2_ and O_3_ per 10 ppb, LAC per 0.5 µg/m^3^), with linearity assessed by comparing linear and natural cubic spline (df = 4) specifications. Sensitivity analyses excluded the 10 participants (0.2%) with suPAR > 20 ng/mL—approximately eight-fold the cohort median—to confirm that estimates were not driven by a small number of extreme values. All analyses were conducted in R 4.5.3 (R Foundation for Statistical Computing, Vienna, Austria).

## Results

Of 6,814 MESA participants, 1,416 lacked a serum suPAR measurement, and 161 lacked air-pollution exposure estimates, leaving a study cohort of 5,237; of these, 5,072 had complete covariate data and were included in the adjusted regression models. The cohort was 52% female, 38% White with a mean age of 63 ± 10 years. PM_2.5_ ranged from 4.4 to 49.4 µg/m^3^ (mean 21.56) and median serum suPAR was 2.46 ng/mL (interquartile range 1.96–3.10) (Table 1). Adjusted PM_2.5_ (1-year average before enrollment) was not associated with suPAR: +0.09% per 5 µg/m^3^ (95% confidence interval −1.34 to +1.54; *P* = 0.90). NO_2_ (−1.91% per 10 ppb; *P* = 0.50), NO_x_ (+2.64% per 40 ppb; *P* = 0.16), and LAC (−0.46% per 0.5 µg/m^3^; *P* = 0.78) were also null. The O_3_ estimate was positive but imprecise (+5.57% per 10 ppb; *P* = 0.08) (Figure 1). Excluding extreme suPAR values (>20 ng/mL; n = 10) attenuated the PM_2.5_ estimate further (−0.14% per 5 µg/m^3^; *P* = 0.84). Departure from linearity for PM_2.5_ was not evident (linear vs. spline, *P* = 0.21).

**Table 1.** Baseline characteristics

Characteristic	MESA cohort (n = 5,237)
Sociodemographic	
Age, years, mean (SD)	62.85 (10.25)
Female, n (%)	2,541 (52)
Race, n (%)	
White	1,999 (38.2)
Chinese	637 (12.2)
Black	1,427 (27.2)
Hispanic	1,174 (22.4)
MESA site, n (%)	
Forsyth County, NC	790 (15.1)
New York, NY	777 (14.8)
Baltimore, MD	887 (16.9)
St. Paul, MN	838 (16.0)
Chicago, IL	890 (17.0)
Los Angeles, CA	1,055 (20.1)
Body mass index, kg/m^2^, mean (SD)	28.28 (5.44)
Neighborhood socioeconomic status score, mean (SD)	−0.25 (1.35)
Education: highest level completed, n (%)	
No schooling	56 (1.1)
Grades 1–8	523 (10.0)
Grades 9–11	366 (7.0)
Completed high-school/GED	963 (18.5)
Some college, no degree	849 (16.3)
Technical school certificate	378 (7.2)
Associate degree	260 (5.0)
Bachelor’s degree	899 (17.2)
Graduate or professional school	922 (17.7)
Cardiovascular risk factors	
Smoking history, n (%)	
Never	2,631 (50.4)
Former	1,936 (37.1)
Current	650 (12.5)
Taking hypertension medication, n (%)	1,991 (38.0)
Systolic blood pressure, mmHg, mean (SD)	127.06 (21.51)
History of diabetes, n (%)	600 (11.5)
Taking statin, n (%)	812 (15.5)
LDL-c, mg/dL, mean (SD)	117.31 (31.71)
HDL-c, mg/dL, mean (SD)	50.91 (14.90)
Estimated glomerular filtration rate, mL/min/1.73m^2^, mean (SD)	77.19 (16.29)
Laboratory values	
suPAR, ng/mL, median (IQR)	2.46 [1.96, 3.10]
Air pollution exposures	
Particulate matter (PM_2.5_), µg/m^3^, mean (SD)^a^	21.56 (9.02)
Nitrogen dioxide (NO_2_), ppb, mean (SD)^a^	20.89 (4.59)
Nitrogen oxides (NO_x_), ppb, mean (SD)^a^	47.64 (23.39)
Ozone (O_3_), ppb, mean (SD)^a^	16.62 (4.51)
Light-absorbing carbon (LAC), µg/m^3^, mean (SD)^b^	0.82 (0.37)

a 1-year average before enrollment.

b Calendar-year 2000 spatial-only baseline average.

IQR indicates interquartile range; SD, standard deviation.

**Figure 1.**
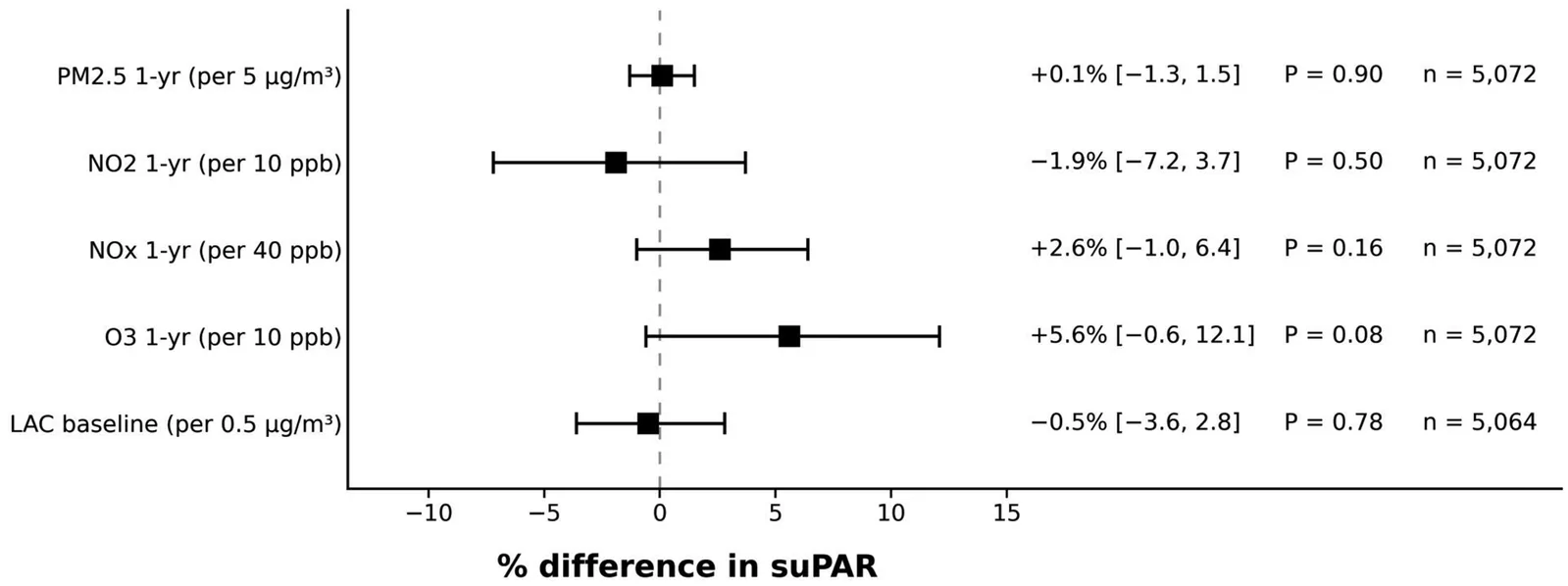
Adjusted percent difference in circulating suPAR per prespecified contrast in long-term ambient air pollution exposure, in 5072 MESA participants. Five exposures shown: PM_2.5_ (1-year average before exam 1, per 5 µg/m^3^), NO_2_ 1-year (per 10 ppb), NO_x_ 1-year (per 40 ppb), O_3_ 1-year (per 10 ppb), and light-absorbing carbon (LAC) baseline (per 0.5 µg/m^3^). Estimates derived from multivariable linear regression of log2(suPAR), adjusted for age, sex, race/ethnicity, MESA site, body mass index, cystatin-C-based estimated glomerular filtration rate, smoking status and pack-years, education, neighborhood socioeconomic status, systolic blood pressure, antihypertensive medication, low- and high-density-lipoprotein cholesterol, statin use, and diabetes. Error bars indicate 95% confidence intervals.

## Discussion

Across a large, multiethnic US cohort, long-term residential ambient air pollution was not associated with circulating suPAR. The PM_2.5_ confidence interval (−1.34 to +1.54% per 5 µg/m^3^) excludes effect sizes ≥2%, a substantially more precise null than either prior report^7,8^ and well below the ~17% increase associated with active smoking.^5^ Three independent cohorts spanning two continents and three age ranges—5,265 Swedish adults (−2.0% per 5 µg/m^3^ PM_2.5_; 95% CI −6.8 to +3.1; converted from the reported natural-log coefficient), 277 US children in whom PM_2.5_ indices were not associated with circulating suPAR, and the present MESA cohort—now converge on the same null, arguing against a meaningful effect of chronic ambient air pollution on suPAR at population-level concentrations.^7,8^ Therefore, suPAR is unlikely to be a major mediator of the air-pollution–cardiovascular-disease link; however, alternative mechanistic pathways (oxidative stress, autonomic dysregulation, and endothelial activation) warrant further investigation beyond the scope of the present work.

Our findings also narrow the search for the modifiable determinants of suPAR, a stable and clinically actionable biomarker.^2^ Notably, cadmium, a chronic accumulating environmental contaminant with renal-cortex half-life measured in decades, has been associated with elevated circulating suPAR in middle-aged adults.^14^ This pattern suggests that suPAR may be responsive to chronic accumulating internal-dose exposures rather than to ambient inhalational exposures at population-level concentrations. Limitations of the present work include cross-sectional design and a single suPAR measurement; however, MESA Air’s spatiotemporal exposure model and 1-year-prior averaging window are optimized for chronic-effect inference and consistent with prior studies.^7,8^ In conclusion, long-term ambient air pollution is not associated with circulating suPAR in a US multiethnic cohort, and the environmental determinants of this clinically actionable biomarker remain to be identified.

## Conflicts of interest statement

S.S.H. has patents pending related to the use of suPAR in the management of patients with cardiovascular disease. The other authors have no conflicts to report.

## ACKNOWLEDGMENTS

The authors thank the participants of the MESA study and the investigators and staff of the MESA Coordinating Center, MESA Air, and the Hayek Laboratory. A full list of participating MESA investigators and institutions is available at https://www.mesa-nhlbi.org.
